# Analgesic Effects and Safety of Dexmedetomidine Added to Nalbuphine or Sufentanil Patient-Controlled Intravenous Analgesia for Children After Tonsillectomy Adenoidectomy

**DOI:** 10.3389/fphar.2022.908212

**Published:** 2022-05-05

**Authors:** Yingping Jia, Rui Zhou, Zhengchen Li, Yuanyuan Wang, Sandong Chen, Liyuan Zhao, Yi Shao, Jinlian Qi

**Affiliations:** Department of Anesthesiology, Children’s Hospital of Henan, Zhengzhou, China

**Keywords:** tonsillectomy, adenoidectomy, patient-controlled intravenous analgesia, dexmedetomidine, nalbuphine, sufentanil

## Abstract

Tonsillectomy is a frequently performed surgical procedure in children, requiring post-operative analgesia. This study evaluated the efficacy and safety of nalbuphine or sufentanil combined with dexmedetomidine for patient-controlled intravenous analgesia (PCIA) after pediatric tonsillectomy adenoidectomy. A total of 400 patients undergoing tonsillectomy with and without adenoidectomy were included in the study. Patients received a PCIA pump (0.5 mg/kg nalbuphine, 2 μg/kg dexmedetomidine and 0.9% sodium chloride to a total volume of 100 ml) for postoperative pain management were classified into Group ND (n = 200). Patients received a PCIA pump (2 μg/kg sufentanil, 2 μg/kg dexmedetomidine and 0.9% sodium chloride to a total volume of 100 ml) for postoperative pain management were classified into Group SD (n = 200). More stable hemodynamic changes were noted in Group ND than Group SD from 1 h to 48 h after operation. At 6, 12, 24, and 48 h after operation, the children in Group ND had higher Ramsay sedation scores than those in Group SD. The times to push the PCIA button in Group ND and Group SD were 2.44 ± 0.74 and 2.62 ± 1.00, showing significant differences (*p* = 0.041). The VASR scores of children in Group ND were significantly lower within 6, 12, and 24 h than those in Group SD (*p* < 0.05). The VASC scores of children in Group ND were significantly lower within four time points (2, 6, 12, and 24 h) than those in Group SD (*p* < 0.05). At 1st day after surgery, the children in Group ND had lower levels of serum ACTH, IL-6, and COR levels than those in Group SD (*p* < 0.001). The incidence rates of nausea and vomiting, and pruritus were significantly higher in Group SD than Group ND (5.00% vs. 11.00%, *p* = 0.028; 1.00% vs. 4.50%, *p* = 0.032). The total incidence rate of adverse reactions was significantly higher in Group SD than Group ND (15.00% vs. 31.00%, *p* = 0.0001). The study demonstrated that dexmedetomidine added to nalbuphine PCIA enhanced the analgesic effects, attenuated the postoperative pain, and reduced the stress response after pediatric tonsillectomy adenoidectomy.

## Introduction

Tonsillectomy with or without adenoidectomy is one of the commonest surgical procedures in children, and recovery from tonsillectomy involves significant and prolonged pain and appears to be more troublesome than other types of childhood surgical procedures (Mitchell et al., 2019). Postoperative pain continues to increase the risk of developing short- and long-term complications, such as delayed behavioral and clinical recovery, including an increased risk of incision bleeding (Batoz et al., 2016). The post-tonsillectomy children may refuse to speak and eat due to intense pain in the throat, which affects nutrition intake and psychological condition, ultimately resulting in poor quality of life (Blackshaw et al., 2020). Moreover, due to the lack of nutrition caused by less eating, the patient’s resistance decreases, increases the chance of postoperative infection, and also affects the healing of postoperative wounds. Well-established principles for successful management of postoperative pain for children include multimodal analgesia, adequate dosage, administration at regular intervals, and use of appropriate route of administration (Krauss et al., 2016; Makhlouf et al., 2019). A variety of analgesic classes, each present their own risk profiles and unique side effects when used for postoperative analgesia in children undergoing tonsillectomy (Cohen and Sommer, 2016). For example, codeine is no longer approved for children aged less than 12 years undergoing tonsillectomy due to obstructive sleep apnea syndrome resulting from clinically relevant polymorphisms in CYP2D6 activity in Europe and United States (Tobias et al., 2016). Paracetamol in combination with a non-steroidal anti-inflammatory drug attenuated the pain after tonsillectomy in children more efficiently (Jotić et al., 2019), while further studies of morphine addition showed mixed results (Kelly et al., 2015; Oremule et al., 2015). Additionally, administration of opioids has been associated with adverse reactions, such as the risk of respiratory depression in case of overdosing and postoperative nausea and vomiting (Kiyatkin, 2019). Postoperative administration of traditional non-steroidal anti-inflammatory drugs such as ketoroic acid and ibuprofen increases the risk of re-operation and bleeding after tonsillectomy (Kokki, 2003). All these represent a major barrier to use in pediatric outpatient surgery. In addition to adverse reactions, parents worries about safety issues around pain medication and refusal of children to take medication make the relief of post-tonsillectomy pain challenging (Lee et al., 2020; Yu and Kim, 2021).

Sufentanil is a newly synthesized *µ*-opioid receptor agonist, which is widely used in many operation and postoperative analgesia due to its strong analgesic effect, long duration, and fewer side effects than morphine (Giaccari et al., 2020). Nalbuphine is a classical opioid receptor agonist-antagonist acting as an agonist of κ-receptors and an antagonist of *µ*-receptors. Nalbuphine has increasingly become a commonly used clinical analgesic drug, especially for the management of visceral pain because of its agonist-antagonist effects and good analgesic effects (Chen et al., 2021). Dexmedetomidine is a selective adrenergic agonist that has less effect on respiratory function, has anti-anxiety, anti-sympathetic nerve and analgesic and sedative properties (Weerink et al., 2017). Previous studies reported the application of dexmedetomidine combined with sufentanil or dexmedetomidine combined with nalbuphine for postoperative analgesia, and both of two postoperative analgesia protocols showing good analgesic effects, reduced doses of sufentanil and nalbuphine, and low incidences of adverse effects (Zhao and Li, 2020; Wang et al., 2022). Patient-controlled analgesia (PCA) is a system that effectively delivers pain relief at a patient’s preferred dose and schedule by allowing them to administer a predetermined bolus dose of medication on-demand at the press of a button (Weibel et al., 2017). PCA has been used since the early 1970s and its application in hospitals has been increasing due to its proven advantages over conventional intramuscular injections (Momeni et al., 2006). PCA has been reported to not only relieve multiple categories of pain, including acute, such as postoperative or labor pain, or chronic, such as palliative care or cancer pain, but also improve pain relief, greater patient satisfaction, less sedation and fewer postoperative complications (Motamed, 2022). Recent evidence has showed the efficacy of patient-controlled intravenous analgesia (PCIA) after pediatric surgery, including pediatric moyamoya surgery (Lim et al., 2020), pediatric thoracotomy for malignancy (Gonzalez et al., 2016), and Nuss surgery (Min et al., 2012). However, limited clinical evidence demonstrated the application of dexmedetomidine combined with sufentanil or dexmedetomidine combined with nalbuphine after pediatric tonsillectomy adenoidectomy. In this study, we analyzed 400 patients undergoing tonsillectomy with and without adenoidectomy who received a PCIA pump (0.5 mg/kg nalbuphine, 2 μg/kg dexmedetomidine and 0.9% sodium chloride to a total volume of 100 ml) for postoperative pain management and a PCIA pump (2 μg/kg sufentanil, 2 μg/kg dexmedetomidine and 0.9% sodium chloride to a total volume of 100 ml) for postoperative pain management, respectively.

## Materials and Methods

### Participants

Pediatric patients undergoing tonsillectomy with and without adenoidectomy at the Children’s Hospital of Henan between January 2018 and June 2021, American Society of anaesthesiologists physical status (ASA-PS) grade I or II, aged 3–6 years were recruited into the study. Children were excluded from enrollment if their parents or guardians were unwilling to receiving postoperative analgesia with PCIA. Children were also excluded for the following reasons: intellectual disability, neurological diseases with agitation-like symptoms, respiratory inhibition diseases or bronchial asthma, cardiac, renal or hepatic disease, severe circulatory system or blood system dysfunction, allergic to study drug administration, body weight >40 kg, or major life changes 1 month before surgery, such as divorce of parents and death of parents. This single center study was approved by the Institutional Review Board of Children’s Hospital of Henan.

### Anaesthesia Protocols

All children fasted for at least 6 h and received no pre-medication before procedure. After the child was transferred to the operating room, non-invasive blood pressure (BP), electrocardiogram, heart rate (HR), pulse oxygen saturation (SpO_2_), end-tidal carbon dioxide partial pressure, and bispectral index (VISTA™ monitoring system, Aspect Medical Systems Inc., Norwood, MA, United States) were continuously monitored. Before anesthesia induction, the child was given intramuscular injection of 0.01 mg/kg atropine and intravenous injection of 1 mg/kg ketamine1 min later. Once consciousness was lost, anesthesia was induced by 2.5 mg/kg propofol, 2 μg/kg fentanyl, and 0.15 mg/kg cisatracurium. Three minutes later, the child was given endotracheal intubation and then mechanically ventilated with volume-controlled ventilation mode aiming at maintain the tidal volume 8–10 ml/kg, the respiratory rate 20 to 25 times/min, oxygen flow 2L/min, the end-tidal carbon dioxide partial pressure at 30–35 mmHg, and the SpO_2_ > 97%. Anesthesia was maintained with 9–15 μg/kg/h propofol and 0.05–0.2 μg/kg/h remifentanil by continuous infusion with a BIS target range of 40–60. Hemodynamic changes were monitored at 5-min intervals throughout the procedure. If systolic blood pressure values decreased 20% below the preoperative baseline value or decreased to 90 mmHg, the child was administered with 10 ml/kg Ringer’s solution and 0.1 mg/kg ephedrine. If the heart rate decreased to 60 beats/min, which was considered bradycardia, and accordingly the child was administered with 0.01 mg/kg atropine.

### Postoperative Analgesia Protocols

All children received PCIA for postoperative pain management with nalbuphine plus dexmedetomidine hydrochloride (Group ND) or sufentanil plus dexmedetomidine hydrochloride (Group SD). At the end of the operation, the child was connected with the wireless analgesic pump system (Rehn Medtech, Jiangsu, China) composed of a patient-controlled analgesia pump transmitting signals, base stations receiving the wireless data, and the monitoring center. The monitoring data from each user are transmitted to the monitoring center in the doctor’s office online. The physicians can then analyze and deal with the data. The PCIA formula for the child allocated into Group ND consisted of nalbuphine 0.5 mg/kg, dexmedetomidine 2 μg/kg and 0.9% sodium chloride to a total volume of 100 ml. The PCIA formula for the child allocated into Group SD consisted of sufentanil 2 μg/kg, dexmedetomidine 2 μg/kg and 0.9% sodium chloride to a total volume of 100 ml. Upon arrival at the general ward, all children and their parents or guardians were instructed on the use of the PCIA pump (Apon, ZZB-I, Medical technology Corporation, Jiangsu, China), and all children were encouraged to push the PCIA button to self-administer their own PCA medications and thus to achieve a rescue analgesic when they were not tolerant to pain throughout 48 h after operation. The PCIA was programmed to deliver a 2 ml bolus on demand, with a lock-out interval of 15 min and a background infusion rate of 2 ml/h. All children were given 2 ml i.v. of PCIA solution immediately after they were attached a PCIA pump. The PCIA was used for the first 48 h postoperatively and thus allowed either a continuous background infusion of nalbuphine 0.01 mg/kg/h with a bolus of nalbuphine 0.005 mg/kg or a continuous background infusion of sufentanil 0.04 μg/kg/h with a bolus of sufentanil 0.02 μg/kg with a continuous background infusion of dexmedetomidine 0.04 μg/kg/h with a bolus of dexmedetomidine 0.02 μg/kg for the child allocated into Group ND and Group SD. The parents received full guidance from anesthesiologists about how to assess pain and push the PCIA button.

### Pain Assessment

Ramsay sedation scores: The Ramsay sedation scale was used to estimate the restlessness scores from 1 to 6 (1, anxious and not reassurable; 2, cooperative, tranquil, and oriented; 3, responds to command; 4, asleep with brisk response to a light glabellar tap or loud auditory stimulus; 5, asleep with sluggish response to a light glabellar tap or loud auditory stimulus; 6, non-responsive). A score of 2–4 indicates good analgesic effect. A score of 5–6 indicates excessive sedation.

Visual analogue score (VAS) at rest (VASR) and at coughing (VASC): VASR was assessed with the child lying supine for static pain intensity and VASC was assessed during change from coughing for dynamic pain intensity. VAS is a 10 cm horizontal “vernier” ranging from 0 to 10 where 0 is defined as no pain, < 3 as sustainable mild pain, 4 to 6 as sustainable but sleep-disturbing pain, and 7 to 10 as unbearable pain to a maximum level of pain. When responding to a VAS item, respondents specify their level of agreement to a statement by indicating a position along a continuous line between two end-points.

### Enzyme Linked Immunosorbent Assay

Fasting venous blood was obtained from each child in the morning on the day before operation, first and second day after operation, respectively. The serum levels of adrenocorticotropic hormone (ACTH), interleukin-6 (IL-6), and cortisol (COR) were detected using ELISA kits (R&D Systems, United States).

### Outcome Variables

Hemodynamic measurements including SBP, DBP, HR and SpO_2_, Ramsay sedation scores, VASR and VASC were recorded at 1, 2, 6, 12, 24, 36, and 48 h after arrival to the ward. The times to push the PCIA button and the incidence of adverse reactions including nausea and vomiting, respiratory depression, cardiovascular events, pruritus and dizziness, were recorded during the studied period. Nausea and vomiting scores were recorded as previously described (Apfel et al., 1999), ranging from 1 to 4 (1, the absence of nausea and vomiting; 2, feel nausea without vomiting; 3, vomiting less than two times; 4, severe vomiting more than two times). Respiratory depression was defined as respiratory depression (ventilatory frequency <10 breath/min lasting for more than 10 min or SpO_2_ < 90%). Cardiovascular events refer to drugrelated bradycardia (HR < 60 beats/min for more than 10 min), and hypotension (>20% decrease in systolic BP or >15% decrease in diastolic BP from preoperative baseline). If severe adverse reactions appeared, the use of PCIA was stopped temporarily and the child was observed continuously for 30 min. If the adverse reactions were presented more than 30 min or a further severe, the child was treated with appropriate medications. Nausea and vomiting were treated with metoclopramide. Respiratory depression was treated with naloxone and oxygen. Hypotension or bradycardia was treated with volume expansion, ephedrine, or atropine. Pruritus was evaluated and treated with diphenhydramine, as appropriate.

### Statistical Analysis

A sample size calculation was performed using PASS 15.0 (NCSS, LLC, Kaysville, Utah, United States) before participant recruitment. Under the supervision of the ethical committee a preliminary trial with 30 pediatric patients under general anesthesia was performed, in which the standard deviation of VASC after tonsillectomy adenoidectomy was found to be 2. With a one-tailed *α* of 0.05 and power of 90%, to gain a difference of no less than 1 in VAS after tonsillectomy adenoidectomy between two equal groups, a total of 86 patients in each group were required. Assuming a dropout rate of 20%, at least 120 children for each group should be recruited in this study.

All statistical analysis was performed Graphpad Prism 8 (GraphPad Software, CA, United States) for Windows. Measurement data are examined by Shapiro Wilk test for normal distribution and shown as mean ± standard deviation and analyzed by paired or unpaired *t* test when normally distributed. Categorical data were shown by number with percentage and analyzed by chi-square test or Fisher’s exact test. The level of *p* < 0.05 reflects the presence of significant difference.

## Results

A total of 400 patients undergoing tonsillectomy with and without adenoidectomy fulfilled the inclusion and excluded criteria were included in the study. The patient characteristics between Group ND (n = 200) and Group SD (n = 200) are summarized in Table 1. There was no difference identified in demographic data including age, gender, weight, ASA-PS grade, duration of operation, length of anesthesia, and transfusion volume between two groups.

**TABLE 1:** Patient characteristics between Group ND and Group SD.

Characteristics	ND (n = 200)	SD (n = 200)	t/Z	*P*
Age (year)	5.04 ± 0.88	5.08 ± 0.83	0.468	0.640
Gender (male/%)	131 (65.50%)	125 (62.50%)	0.625	0.532
Weight (kg)	20.29 ± 4.04	20.31 ± 3.50	0.053	0.958
ASA-PS grade (n)	-	-	0.817	0.414
Grade I	52	45	-	-
Grade II	148	155	-	-
Type of surgery (n)	-	-	0.717	0.474
Tonsillectomy	82	75	-	-
Tonsillectomyandadenoidectomy	118	125	-	-
Duration of operation (min)	35.72 ± 16.83	34.42 ± 15.11	0.813	0.417
Length of anesthesia (min)	44.21 ± 20.18	42.89 ± 18.37	0.684	0.494

ASA-PS, American Society of anaesthesiologists physical status; Data of mean ± standard deviation were analyzed by unpaired *t* test and data of number with percentage were analyzed by chi-square test or Fisher’s exact test.

**FIGURE 1:**
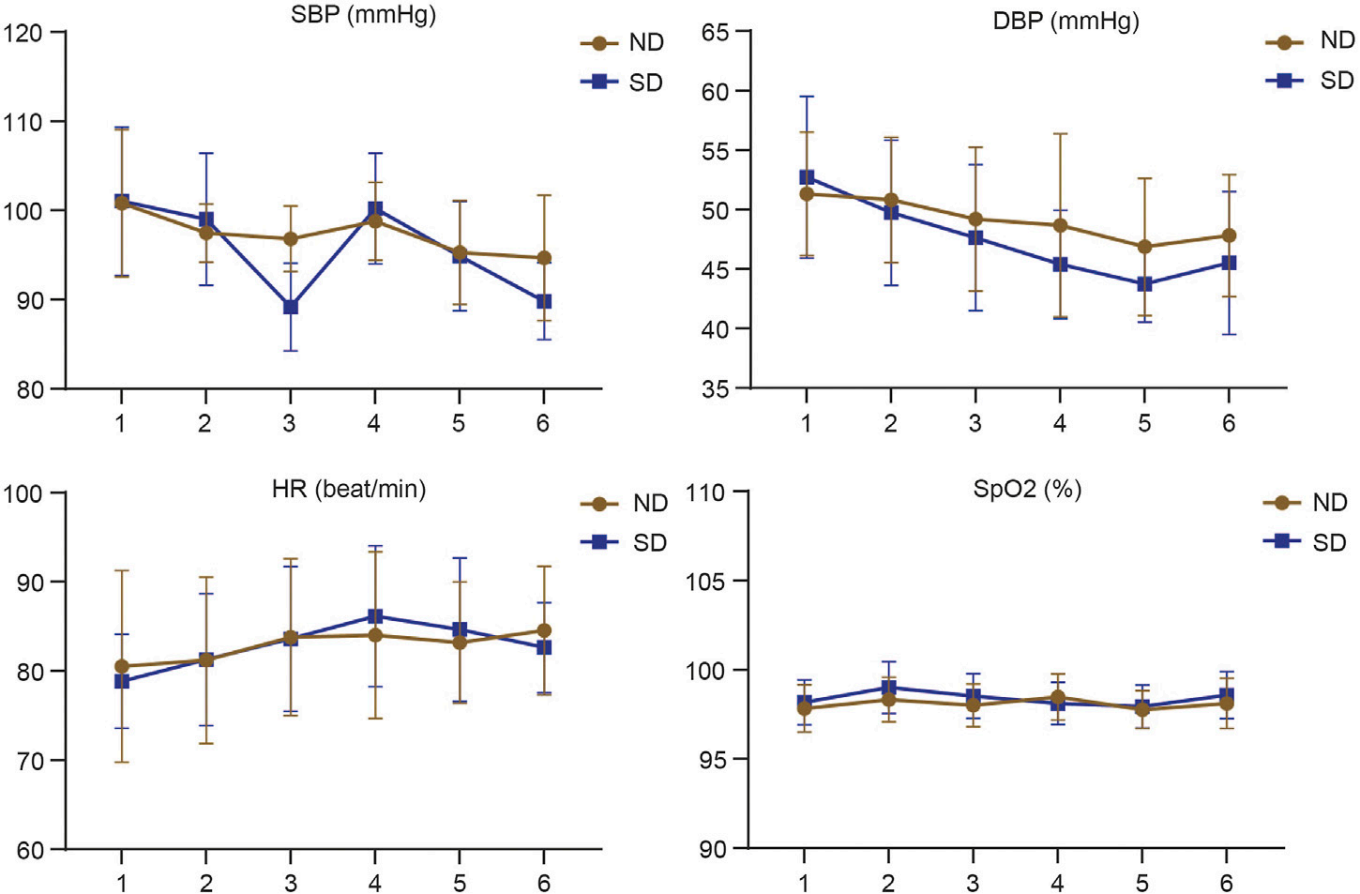
Hemodynamic changes assessed by SBP, DBP, HR and SpO_2_ of children at 1, 2, 6, 12, 24, and 48 h after operation between Group ND and Group SD.

Hemodynamic measurements including SBP, DBP, HR and SpO_2_ at 1, 2, 6, 12, 24, and 48 h after arrival to the ward were shown in Table 2. With regard to SBP, the children in Group SD exhibited significant changes at indicated time points, while those in Group ND exhibited significant changes only at 2, 12, and 24 h after surgery (*p* < 0.05). As for DBP, no evidence of hypotension was found in each group, while the children in Group SD exhibited significant changes in DBP among each time points (*p* < 0.05). The children in Group ND exhibited no significant change at each time point (*p* > 0.05). It was found that the children in Group ND exhibited significant changes in HR only at 6 h after surgery (*p* < 0.05), but those in Group SD exhibited significant changes at indicated time points (*p* < 0.05). No remarkable changes were found in both groups at indicated time points (*p* > 0.05) after surgery. These data suggested that more stable hemodynamic changes were noted in pediatric patients receiving nalbuphine combined with dexmedetomidine than sufentanil combined with dexmedetomidine for PCIA (Figure 1).

**TABLE 2:** Hemodynamic measurements of children at 1, 2, 6, 12, 24, and 48 h after arrival to the ward between Group ND and Group SD.

Hemodynamic Measurements	ND (n = 200)	SD (n = 200)
SBP (mmHg)		
1 h after surgery	100.83 ± 8.27	101.04 ± 8.32
2 h after surgery	97.48 ± 3.25*	99.02 ± 7.41*
6 h after surgery	96.83 ± 3.68	89.17 ± 4.92*
12 h after surgery	98.79 ± 4.36*	100.20 ± 6.20*
24 h after surgery	95.30 ± 5.82*	94.88 ± 6.12*
48 h after surgery	94.68 ± 7.04	89.83 ± 4.33*
DBP (mmHg)		
1 h after surgery	51.34 ± 5.19	52.74 ± 6.80*
2 h after surgery	50.83 ± 5.27	49.76 ± 6.11*
6 h after surgery	49.20 ± 6.05	47.64 ± 6.14*
12 h after surgery	48.68 ± 7.70	45.41 ± 4.56*
24 h after surgery	46.88 ± 5.76	43.75 ± 3.22*
48 h after surgery	47.83 ± 5.13	45.53 ± 6.01*
HR (beat/min)		
1 h after surgery	80.53 ± 10.76	78.86 ± 5.27
2 h after surgery	81.22 ± 9.33	81.29 ± 7.40*
6 h after surgery	83.80 ± 8.79*	83.61 ± 8.12*
12 h after surgery	84.02 ± 9.35	86.14 ± 7.90*
24 h after surgery	83.17 ± 6.80	84.65 ± 8.03
48 h after surgery	84.54 ± 7.22	82.64 ± 5.05*
SpO_2_ (%)		
1 h after surgery	97.83 ± 1.33	98.17 ± 1.26
2 h after surgery	98.33 ± 1.25	99.00 ± 1.46
6 h after surgery	98.01 ± 1.20	98.53 ± 1.25
12 h after surgery	98.47 ± 1.29	98.11 ± 1.18
24 h after surgery	97.76 ± 1.06	97.94 ± 1.21
48 h after surgery	98.11 ± 1.41	98.57 ± 1.32

* indicates *p* < 0.05 by paired *t* test in comparison with the previous time point.

Ramsay sedation scores of children at 1, 2, 6, 12, 24, and 48 h after arrival to the ward between Group ND and Group SD were listed in Table 3. No evidence of distinct difference was found regarding Ramsay sedation scores of children between Group ND and Group SD at 1 and 2 h after surgery (*p* > 0.05). At 6, 12, 24, and 48 h after surgery, the children allocated into Group ND had higher Ramsay sedation scores than those allocated into Group SD (*p* < 0.05). No case with excessive sedation (Ramsay sedation scores ≥5) was observed. These data revealed a more analgesic effect conferred by nalbuphine combined with dexmedetomidine than sufentanil combined with dexmedetomidine for PCIA.

**TABLE 3:** Ramsay sedation scores of children at 1, 2, 6, 12, 24, and 48 h after arrival to the ward between Group ND and Group SD.

	ND (n = 200)	SD (n = 200)	t	*P*
1 h after surgery	3.31 ± 0.50	3.26 ± 0.45	1.051	0.294
2 h after surgery	3.26 ± 0.48	3.18 ± 0.52	1.599	0.111
6 h after surgery	3.17 ± 0.47	3.02 ± 0.40	3.437	0.006
12 h after surgery	3.08 ± 0.41	2.99 ± 0.29	2.534	0.012
24 h after surgery	2.32 ± 0.57	2.19 ± 0.23	2.991	0.003
48 h after surgery	2.05 ± 0.50	1.93 ± 0.15	3.251	0.001

Statistical analysis was performed by unpaired *t* test.

The times to push the PCIA button in Group ND and Group SD were 2.44 ± 0.74 and 2.62 ± 1.00, showing significant difference between two groups (t = 2.046, *p* = 0.041).

VAS scores of children at 1, 2, 6, 12, 24, and 48 h after operation between Group ND and Group SD were shown in Table 4. There was no significant difference of VASR scores within 1 and 2 h post operation and of VASC scores within 1 h post operation between the two groups (*p* > 0.05). The VASR scores of children in Group ND were significantly lower within 6, 12, and 24 h than those in Group SD (*p* < 0.05). The VASC scores of children in Group ND were significantly lower within four time points (2, 6, 12, and 24 h) than those in Group SD (*p* < 0.05). No significant difference regarding both scores within 48 h post operation between the two groups (*p* > 0.05). These results indicated that nalbuphine combined with dexmedetomidine in PCIA could relieve the postoperative pain compared with sufentanil combined with dexmedetomidine in PCIA for pediatric tonsillectomy adenoidectomy.

**TABLE 4:** VAS scores of children at 1, 2, 6, 12, 24, and 48 h after arrival to the ward between Group ND and Group SD.

VAS Scores	ND (n = 200)	SD (n = 200)	t	*P*
VASR				
1 h after surgery	2.36 ± 0.52	2.44 ± 0.58	1.452	0.147
2 h after surgery	2.50 ± 0.57	2.60 ± 0.60	1.709	0.088
6 h after surgery	2.81 ± 0.59	3.01 ± 0.63	3.277	0.001
12 h after surgery	3.06 ± 0.50	3.20 ± 0.55	2.644	0.008
24 h after surgery	2.86 ± 0.49	2.98 ± 0.53	2.351	0.019
48 h after surgery	2.29 ± 0.43	2.35 ± 0.45	1.363	0.174
VASC				
1 h after surgery	2.55 ± 0.60	2.62 ± 0.62	1.147	0.252
2 h after surgery	2.88 ± 0.57	3.03 ± 0.60	2.563	0.011
6 h after surgery	3.27 ± 0.66	3.53 ± 0.68	3.880	0.001
12 h after surgery	3.60 ± 0.66	3.78 ± 0.68	2.686	0.008
24 h after surgery	3.39 ± 0.60	3.52 ± 0.63	2.113	0.035
48 h after surgery	2.62 ± 0.57	2.72 ± 0.60	1.709	0.088

Statistical analysis was performed by unpaired *t* test.

**FIGURE 2:**
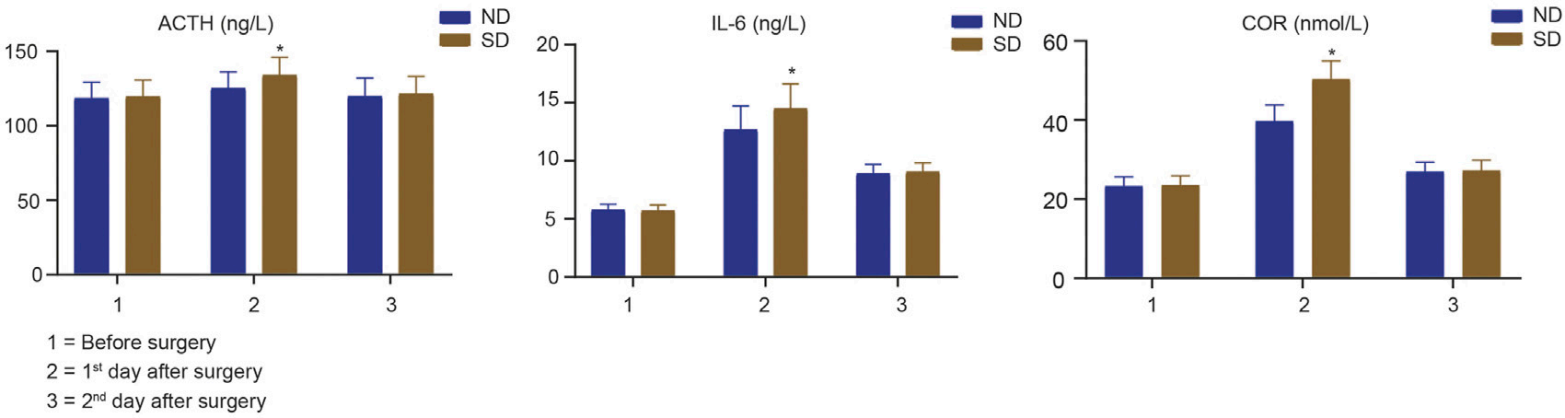
The serum ACTH, IL-6, COR levels in children first day before surgery, first and second day after operation between Group ND and Group SD were determined by ELISA methods. * indicates *p* < 0.05.

The ACTH, IL-6, COR levels in the serum extracted from each child first day before surgery, first and second day after surgery between Group ND and Group SD were determined by ELISA methods. As shown in Figure 2, two groups exhibited no significant difference with regard to serum levels of ACTH, IL-6, COR in children first day before surgery (*p* > 0.05). At 1st day after surgery, the children in Group ND had lower levels of serum ACTH (t = 6.516, *p* < 0.001), IL-6 (t = 8.685, *p* < 0.001), and COR (t = 23.880, *p* < 0.001) levels than those in Group SD. At 2nd day after surgery, no significant difference was noted in serum levels of ACTH, IL-6, and COR in children between two groups (*p* > 0.05). These data suggested that nalbuphine combined with dexmedetomidine in PCIA could reduce stress response of pediatric patients undergoing tonsillectomy adenoidectomy.

The children may suffer several adverse reactions, including nausea and vomiting, dizziness and headache, respiratory depression, dry mouth, restlessness, and pruritus. As shown in Table 5, no child suffered respiratory depression. There were 10 cases of nausea and vomiting, 8 cases of dizziness and headache, 7 cases of dry mouth, 3 cases of restlessness, and 2 cases of pruritus in Group ND. There were 22 cases of nausea and vomiting, 12 cases of dizziness and headache, 12 cases of dry mouth, 7 cases of restlessness, and 9 cases of pruritus in Group SD. The incidence rates of nausea and vomiting, and pruritus were significantly higher in Group SD than Group ND (5.00% vs. 11.00%, *p* = 0.028; 1.00% vs. 4.50%, *p* = 0.032), while the incidence rates of dizziness and headache, dry mouth, and restlessness were not (*p* > 0.05). The total incidence rate of adverse reactions was significantly higher in Group SD than Group ND (15.00% vs. 31.00%, *p* = 0.0001). These data revealed the safety of nalbuphine combined with dexmedetomidine in PCIA for postoperative pain management in pediatric tonsillectomy adenoidectomy.

**TABLE 5:** The incidence of adverse reactions in children receiving PCA between Group ND and Group SD.

Group	Nausea and vomiting	Dizziness and headache	Respiratory depression	Dry mouth	Restlessness	Pruritus	Total
ND (n = 200)	10 (5.00%)	8 (4.00%)	0	7 (3.50%)	3 (1.50%)	2 (1.00%)	30 (15.00%)
SD (n = 200)	22 (11.00%)	12 (6.00%)	0	12 (6.00%)	7 (3.50%)	9 (4.50%)	62 (31.00%)
*P*	0.028	0.358	0	0.240	0.200	0.032	0.0001

Statistical analysis was performed by chi-square test or Fisher’s exact test.

## Discussion

Alleviating the pain after tonsillectomy in children has always been a challenge and the choice of postsurgical analgesic agents remains controversial. Opioid analgesics are widely used in PCIA in clinic, including fentanyl, morphine, and sufentanil. Although these analgesics present good analgesic effect, they may be associated with various adverse reactions such as respiratory depression especially for children with obstructive sleep apnea (Chidambaran et al., 2017), excessive sedation, blood pressure drop, gastrointestinal peristalsis, nausea and vomiting, skin pruritus, urinary retention (Sadhasivam et al., 2015). The use of alternative nonsteroidal anti-inflammatory drugs such as ketorolac and ibuprofen contributes to the increased risk of hemorrhage after tonsillectomy (Møiniche et al., 2003; Tan and Tunkel, 2017). Naborphine, as a new type of synthetic opioid, has emerged its clinical value in recent years. Nalbuphine is a powerful analgesic showing low side effects and low dependence in animals and humans, and it has been shown to be effective against the respiratory inhibitory without attenuation of analgesia (Klepper et al., 1986; Narver, 2015; Haw et al., 2016). Previous studies have confirmed that combination of dexmedetomidine and sufentanil on spinal anesthesia (Karimi et al., 2021) or nalbuphine combined with dexmedetomidine in the postoperative treatment of laparoscopic oophorocystectomy (Liu et al., 2021) achieved good clinical effects such as longer postoperative analgesia, reduced use of analgesic, and slight hemodynamic changes. This study attempted to investigate the impacts of sufentanil or nalbuphine combined with dexmedetomidine on the children after tonsillectomy.

Under the condition of PCIA, drugs can be infused according to the specified time and concentration, leading to lower dosage, improvements on the efficacy and safety of drugs, and decrease in side effects. PCIA has been proved to be an excellent method of postoperative pain relief in adults and children undergoing surgical intervention (Morlion et al., 2018). In this study, we adopted PCIA method with different analgesics for the patients who underwent tonsillectomy for postoperative pain management. Perioperative haemodynamic monitoring is the cornerstone of optimizing tissue perfusion and preventing metabolic deterioration. Hemodynamic instability is related to cardiac dysfunction (Pang et al., 2019). Dexmedetomidine inhibits the release of norepinephrine through stimulating presynaptic and postsynaptic *α* 2 receptors, which in turn slows the patient’s heart rate and reduces blood pressure, and then maintain the stability of hemodynamics of patients (Weerink et al., 2017). The present study found that the children received sufentanil and dexmedetomidine showed significant changes regarding SBP at time points including 1, 2, 6, 12, 24, and 48 h after arrival to the ward, and the children treated with nalbuphine and dexmedetomidine had significant changes of SBP only at 2, 12, and 24 h after arrival. The two groups presented the absence of hypotension. However, significant changes in DBP at each time points were found in the Group SD but not in the Group ND. As for the HR, the Group SD exhibited significant changes at all points while the Group ND revealed significant changes only at 6 h after arrival. The two groups showed slight changes in SpO_2_ at indicated time points after arrival. These findings suggested that the children had more stable hemodynamics after administration of nalbuphine and dexmedetomidine. Naborphine is a new synthetic opioid drug, which belongs to agonist-antagonist drugs. It exerts analgesic effect mainly through stimulation of k receptor and it has significant antagonism to *µ* receptor, which relatively reduces adverse reactions induced by *µ* receptor-related opioids such as respiratory depression, nausea and vomiting, skin pruritus, and hemodynamic instability (Wu et al., 2021). Etches (1994) indicated that the incidence of respiratory depression is 0.1%–1% regardless of the route of administration of opioids, and the degree of respiratory depression is associated with dose. This study confirmed that no patient had respiratory depression in the two groups, which might be benefited from the use of dexmedetomidine resulting in decrease use of opioids. The two groups showed no significant difference in the incidence rates of dizziness and headache, dry mouth, and restlessness. However, Group ND revealed significantly lower incidence rates of nausea and vomiting, and pruritus than the Group SD. These outcomes were indirectly supported by other studies indicating the addition of nalbuphine in parturients undergoing cesarean delivery contributed to lower incidence of postoperative adverse reactions including nausea, vomiting and pruritus (Ibrahim et al., 2019). Tubog et al. (2019) also proved that reduced incidence of pruritus induced by neuraxial opioids was relevant with the use of nalbuphine.

Postoperative pain experience will have a long-term impact on children, such as long-term behavioral changes and reduced pain tolerance, which will directly affect the development and growth of children’s emotional and activity ability in the future (Howard, 2003). Therefore, it is very important to do a good job in perioperative analgesia. In this study, it was observed that the two groups showed slight difference in Ramsay sedation scores at 1 and 2 h after arrival to the ward. However, the Group ND had higher Ramsay sedation scores at 6, 12, 24, and 48 h after arrival than the Group SD, and there was no one with Ramsay sedation scores ≥5 in the two groups. The results demonstrated the analgesic effect of naborphine combined with dexmedetomidine is better than that of sufentanil combined with dexmedetomidine. VAS is a scale developed to obtain more variable measurements. It uses linear continuum to measure potential features and is widely used to measure postoperative pain intensity (Sung and Wu, 2018). We compared VAS scores of each groups at different time points including 1, 2, 6, 12, 24, and 48 h after operation. The Group ND presented significantly lower VASR scores (6, 12, 24 h) and VASC scores (2, 6, 12, and 24 h) than the Group SD. Previous study of orthognathic surgery revealed that the patients who received naborphine had significant lower VAS scores, and higher Ramsay scores than the patients who treated with sufentanil (Xi et al., 2020). The results indirectly confirmed our above findings.

Surgical trauma can also cause various body reactions, including activation of leukocytes and synthesis of cell factors. Cytokines are involved in a variety of reaction processes *in vivo*, and IL-6 is an important pro-inflammatory cytokine in body reactions (Jawa et al., 2011; Yuan, 2018). Pro-inflammatory cytokine release promotes phagocytosis of macrophages to remove invading pathogens and necrotic tissue cells. However, its overexpression will cause the expansion of inflammation, which is not conducive to the recovery of the body. Dexmedetomidine, as a α2 adrenoceptor agonist, has been shown to reduce systemic inflammation induced by sepsis *in vivo* (Sun et al., 2019) and *in vitro* (Mei et al., 2021). In our study, at first day after surgery, lower levels of serum IL-6 was found in the Group ND than that in the Group SD, and slight difference was observed at second day after surgery between two groups. Liu et al. manifested naborphine with high dose (1.5 mg/kg) plus dexmedetomidine (4 μg/kg) was beneficial to reduce IL-6 levels at first day after laparoscopic oophorocystectomy (Liu et al., 2021). The present study also confirmed that nalbuphine combined with dexmedetomidine significantly reduced levels of ACTH and COR at the first day after surgery, which suggested this administration could reduce stress response after tonsillectomy.

Several study limitations should be noted when interpreting our data. First, included surgical and anesthetic protocols were done by several surgeons and anesthesiologists. Although they all had the same experience with pediatric tonsillectomy adenoidectomy and anaesthesia, bias on surgical outcomes may be considered. Second, due to retrospective nature, our data were obtained through past records, which may introduce potential bias by patient selection. Third, we investigated only one dose of study drugs, and a dose-response study was not performed. Thus, there might be a better dose for a more effective combination, and further prospective studies will be required to evaluate dose-dependent effects. Fourth, we failed to analyze pediatric patients receiving PCIA of single dose of nalbuphine, sufentanil or dexmedetomidine and compared them with combination groups. Fifth, this was a single-center study, and thus more high-quality, multi-center, large-simple randomized trials are still warranted to optimize PCIA protocols for being more practical in clinical practice.

The results of this study revealed that in PCIA mode, naborphine combined with dexmedetomidine presented better clinical efficacy than the combination of dexmedetomidine and sufentanil after tonsillectomy. Dexmedetomidine and naborphine can further enhanced analgesic effect, maintain hemodynamics, reduce the incidence of nausea and vomiting, and decrease stress response.

## Data Availability

The original contributions presented in the study are included in the article/Supplementary Material, further inquiries can be directed to the corresponding author.
